# Near-infrared fluorescence imaging and photodynamic therapy with indocyanine green lactosome has antineoplastic effects for hepatocellular carcinoma

**DOI:** 10.1371/journal.pone.0183527

**Published:** 2017-08-31

**Authors:** Takumi Tsuda, Masaki Kaibori, Hidehiko Hishikawa, Richi Nakatake, Tadayoshi Okumura, Eiichi Ozeki, Isao Hara, Yuji Morimoto, Kengo Yoshii, Masanori Kon

**Affiliations:** 1 Department of Surgery, Kansai Medical University, Hirakata, Osaka, Japan; 2 Technology Research Laboratory, Shimadzu Corporation, Kyoto, Japan; 3 Department of Integrative Physiology and Bio-Nano Medicine, National Defense Medical College, Tokorozawa, Saitama, Japan; 4 Department of Mathematics and Statistics in Medical Sciences, Kyoto Prefectural University of Medicine, Kyoto, Japan; Massachusetts General Hospital, UNITED STATES

## Abstract

**Background:**

Anticancer agents and operating procedures have been developed for hepatocellular carcinoma (HCC) patients, but their prognosis remains poor. It is necessary to develop novel diagnostic and therapeutic strategies for HCC to improve its prognosis. Lactosome is a core-shell-type polymeric micelle, and enclosing labeling or anticancer agents into this micelle enables drug delivery. In this study, we investigated the diagnostic and therapeutic efficacies of indocyanine green (ICG)-loaded lactosome for near-infrared fluorescence (NIF) imaging and photodynamic therapy (PDT) for HCC.

**Methods:**

The human HCC cell line HuH-7 was treated with ICG or ICG-lactosome, followed by PDT, and the cell viabilities were measured (*in vitro* PDT efficiency). For NIF imaging, HuH-7 cells were subcutaneously transplanted into BALB/c nude mice, followed by intravenous administration of ICG or ICG-lactosome. The transplanted animals were treated with PDT, and the antineoplastic effects were analyzed (*in vivo* PDT efficiency).

**Results:**

PDT had toxic effects on HuH-7 cells treated with ICG-lactosome, but not ICG alone. NIF imaging revealed that the fluorescence of tumor areas in ICG-lactosome-treated animals was higher than that of contralateral regions at 24 h after injection and thereafter. PDT exerted immediate and continuous phototoxic effects in the transplanted mice treated with ICG-lactosome.

**Conclusions:**

Our results demonstrate that ICG-lactosome accumulated in xenograft tumors, and that PDT had antineoplastic effects on these malignant implants. NIF imaging and PDT with ICG-lactosome could be useful diagnostic and/or therapeutic strategies for HCC.

## Introduction

Worldwide, hepatocellular carcinoma (HCC) is the fifth and eighth most common malignancy in men and women, respectively, and more than 500,000 new cases are diagnosed each year [1,2]. HCC generally has a poor prognosis; the 5-year survival rate for hepatic tumors is 33.5% for males and 30.5% for females in Japan [3]. Although a variety of anticancer agents and operating procedures have been developed in recent years, they have proven insufficient to improve the overall prognosis. For example, sorafenib is an orally administered multi-kinase inhibitor that is used to treat patients with advanced HCC [4]; however, the therapeutic effects of sorafenib are limited. The strong side effects of sorafenib prevent long-term treatment of patients with advanced HCC. The long-term prognosis of HCC patients who are treated with curative intent is unsatisfactory, and the efficacies of available chemotherapeutic agents are limited [4–6]. Thus, novel therapies are required to improve the prognosis of refractory HCC.

The photosensitizing agent indocyanine green (ICG) is a water-soluble tricarbocyanine dye that emits fluorescence with a peak wavelength of 830 nm when illuminated with near-infrared excitation light [7]. In clinical settings, ICG is used to identify sentinel lymph node metastases in breast cancer, ophthalmic angiography, and coronary artery blood flow evaluation. In the field of gastrointestinal surgery, it is used for liver function evaluation and intraoperative local diagnosis of HCC by fluorescence imaging techniques [7–10]. Although intravenously-injected ICG is exclusively excreted in the bile, ICG accumulates in hepatic tumor tissues because of bile excretion disorders in HCC. When the liver is illuminated with near-infrared excitation light, retained ICG emits fluorescence and thus HCC can be detected [8]. The stability of ICG accumulated in tumor tissues depends on the degree of tumor differentiation and normal tissue fibrillization around the tumor [8,11].

Recently, ICG has been used for photodynamic therapy (PDT), which utilizes a photochemical reaction between photosensitizing agents and laser light of a specific wavelength. Briefly, a photosensitizing agent is administered and accumulates in cancer tissues, and is then irradiated with laser light directed onto the tumor. The activated photosensitizing agent reacts with endogenous oxygen, resulting in the generation of reactive oxygen species (ROS), such as singlet oxygen and free radicals, which lead to cell death processes, such as apoptosis, in tumor cells. In addition, heat is generated by the reaction, and this thermal effect contributes to the tumor-suppressive effect [9,12]. PDT has been primarily applied as a local therapy for malignancies such as skin cancer [13] and superficial bladder cancer [14]. However, it has recently become more widely accepted as a treatment option for early gastric, esophageal, and lung cancer [15–17].

Nanocarriers of polymeric micelles have potential applications for both tumor imaging and antitumor therapy. These micelles accumulate in solid tumors through the enhanced permeability and retention (EPR) effect. The EPR effect is a phenomenon wherein 10–200-nm nanoparticles passively accumulate in tumors because of the hyperpermeability of tumor vessels and the lowered lymph flow around tumor tissues [18]. However, nanocarriers are likely to be captured by cells of the hepatic reticuloendothelial system (RES) and accumulate in the liver. Therefore, it is difficult to selectively identify their accumulation in liver tumors. Lactosome is a core-shell-type nanocarrier, comprising a polymeric micelle composed of amphiphilic polydepsipeptide with a hydrophobic block of helical poly (_L_-lactic acid) (PLLA) and a hydrophilic block of poly (sarcosine) (PS). Lactosome is a biocompatible and biodegradable material that has no acute toxicity, and can selectively accumulate in liver tumors, by avoiding the RES [19–22].

These observations prompted us to develop a drug delivery system against HCC. In this study, we prepared an ICG-containing lactosome, termed ICG-lactosome, that accumulated in a tumor-specific manner, and investigated this compound for its diagnostic and therapeutic efficacies in PDT for HCC.

## Materials and methods

### Cancer cell lines

HuH-7 cells (a well-differentiated human HCC cell line) were purchased from the Japanese Collection of Research Bioresources (JCRB) Cell Bank (Osaka, Japan). Cells were suspended in culture medium and incubated at 37°C in an incubator under a humidified atmosphere containing 5% CO_2_. The culture medium was Dulbecco’s modified Eagle’s medium (Wako, Osaka, Japan) supplemented with 10% heat-inactivated fetal bovine serum (Thermo Fisher Scientific, Waltham, MA, USA), penicillin (100 U/mL), streptomycin (100 μg/mL), and amphotericin B (0.25 μg/mL) (Antibiotic-Antimycotic Mixed Stock Solution; Nacalai Tesque, Kyoto, Japan).

### ICG-lactosome

The lactosome used was a micelle assembled from block copolymers of PS and PLLA (PS—PLLA) that were synthesized as previously reported [20]. As PS—PLLA is composed of a biodegradable polypeptide, its toxicity is negligible, thus suggesting its safe use in humans. The ICG-lactosome prepared in this study contained 22% ICG—PLLA and 78% PS—PLLA. The ICG—PLLA was synthesized as previously reported [23]. Chloroform solutions of PS—PLLA and ICG—PLLA were mixed at a ratio of 0.78:0.22. The solvent was removed under reduced pressure and the formed thin film was dissolved in 10 mM Tris—HCl buffer (pH 7.4). The resulting aqueous solution was purified by Sephacryl S-100 size-exclusion chromatography with elution by 10 mM Tris—HCl buffer (pH 7.4) to obtain ICG-lactosome. Dynamic light-scattering analysis revealed that the hydrodynamic diameter of ICG-lactosome was 40–50 nm.

### *In vitro* PDT

HuH-7 cells were seeded into 96 multiwell plates (BD Falcon, Tokyo, Japan) at 2×10^4^ cells/100 μL culture medium/well and incubated for 24 h. The plates were divided into four groups (4 wells/group): cells without ICG-lactosome or laser light (control group); cells without ICG-lactosome but with laser light (laser group); cells with ICG-lactosome but without laser light (ICG-lactosome group); and cells with ICG-lactosome and laser light (PDT group). On the next day, the medium was changed as follows: phosphate-buffered saline (PBS(-)) for the control and laser groups; and PBS(-) containing 2 mg/mL ICG-lactosome for the ICG-lactosome and PDT groups. The laser and PDT groups were irradiated using a near-infrared (810±10 nm) laser source (AVL-20; Asuka Medical, Kyoto, Japan) immediately after changing the culture medium. The fluence rates (mW/cm^2^) and irradiation periods (s) of the laser were set in four ways, 190 mW/cm^2^ and 95 s, 340 mW/cm^2^ and 55 s, 190 mW/cm^2^ and 525 s, and 340 mW/cm^2^ and 300 s, corresponding to a fluence of approximately 18 J/cm^2^ or 100 J/cm^2^. The laser probe was set at 2 cm above the plate. During irradiation, we measured the temperature of the medium using a thermo-camera (Testo 890; Testo K.K., Kanagawa, Japan). After irradiation, each well was changed to fresh culture medium and incubated for 0, 24, 48, 72, or 96 h.

### Assaying cell viability after irradiation

Cell morphology was observed with a phase-contrast microscope, and cell viability after irradiation was measured by the MTT assay (Cayman Chemical, Ann Arbor, MI, USA). Briefly, 10 μL of MTT reagent was added to each well and incubated for 3 h at 37°C in a CO_2_ incubator, followed by aspiration of the medium, addition of 100 μL of Crystal Dissolving Solution to dissolve the formazan crystals, and measurement of the absorbance at 570 nm using a microplate reader.

### Animals and ethics

Male BALB/c nude mice (5 weeks-old) were purchased from Shimizu Laboratory Supplies Co. Ltd. (Kyoto, Japan). The mice were kept at 22°C under a 12-h/12-h light/dark cycle, and received food and water *ad libitum*. All animal experiments were performed in accordance with the Guidelines for the Care and Use of Laboratory Animals of the National Institutes of Health, and approved by the Animal Care Committee of Kansai Medical University.

### Animal models

Mice were anesthetized with isoflurane and subcutaneously injected into the left inguinal region with 5×10^6^ HuH-7 cells in 100 μL of culture medium. When the tumors reached 50–1000 mm^3^ (approximately 3 weeks after transplantation), the experiments with *in vivo* imaging or PDT were terminated. Mice were euthanized when the major tumor size exceeded 2 cm. No animals died from tumors below 2 cm in size in this experiment. The mice were observed at an average frequency of once every 2 days.

### *In vivo* imaging

Model mice were anesthetized with isoflurane and intravenously injected through the tail vein with 100 μL of 20 mg/mL ICG-lactosome containing 0.2 mg/mL ICG (ICG-lactosome group, n = 5) or 100 μL of 0.2 mg/mL ICG (ICG group, n = 5) dissolved in saline. After administration, mice were anesthetized with isoflurane and imaged at 0, 3, 6, 24, 48, 72, and 168 h. *In vivo* fluorescence imaging was acquired using an IVIS system (PerkinElmer, Waltham, MA, USA). Engrafted tumors were illuminated with a 780-nm excitation light, and the fluorescence was acquired using an 845-nm filter. The brightness of the tumor and non-tumor (contralateral inguinal) areas was measured on the obtained fluorescence images and recorded.

### *In vivo* PDT

Model mice were anesthetized with isoflurane and intravenously injected through the tail vein with 200 μL of 20 mg/mL ICG-lactosome containing 0.2 mg/mL ICG (ICG-lactosome group, n = 6) or 200 μL 0.2 mg/mL ICG (ICG group, n = 6). Irradiation using the near-infrared laser source (AVL-15) was performed at 48 h after injection, with the output of the fiber probe placed at 1 cm above the xenograft tumors. The fluence rate was set at 500 mW/cm^2^, and the irradiation period was set at 200 s (100 J/cm^2^). Compared with the *in vitro* setup, we raised the fluence rate to 500 mW/cm^2^, because it was not initially possible to obtain a sufficient temperature increase with the low fluence rate set by reference to a previous report [23]. The temperature of the tumors in mice treated with PDT was measured by the Testo 890 thermo-camera. After treatment, the tumor volumes were measured every 2 days until day 8. Tumor volumes were calculated by the formula: (major axis × minor axis^2^)/2.

### Pathology

Pathological specimens were collected for examination of apoptosis. Tumors were obtained before and at 24 h after irradiation (*in vivo* PDT) in the ICG and ICG-lactosome groups. Apoptosis was observed by TdT-mediated dUTP nick-end labeling (TUNEL). The apoptotic index was calculated as the ratio of positively-stained cells to the total number of tumor cells in each case. To determine the apoptotic index, five representative areas comprising at least 1000 cells without inflammation or necrosis were counted for each sample using a light microscope at 400× magnification.

### Statistical analysis

Data are presented as mean ± standard deviation. Differences between groups were assessed by two-way analysis of variance and the Tukey—Kramer post hoc test. The apoptotic index was assayed by Student’s *t*-test. The level of significance was set at P<0.05. All statistical analyses were performed with R version 3.0.2 (R Foundation for Statistical Computing, Vienna, Austria).

## Results

### *In vitro* PDT

**Fig 1:**
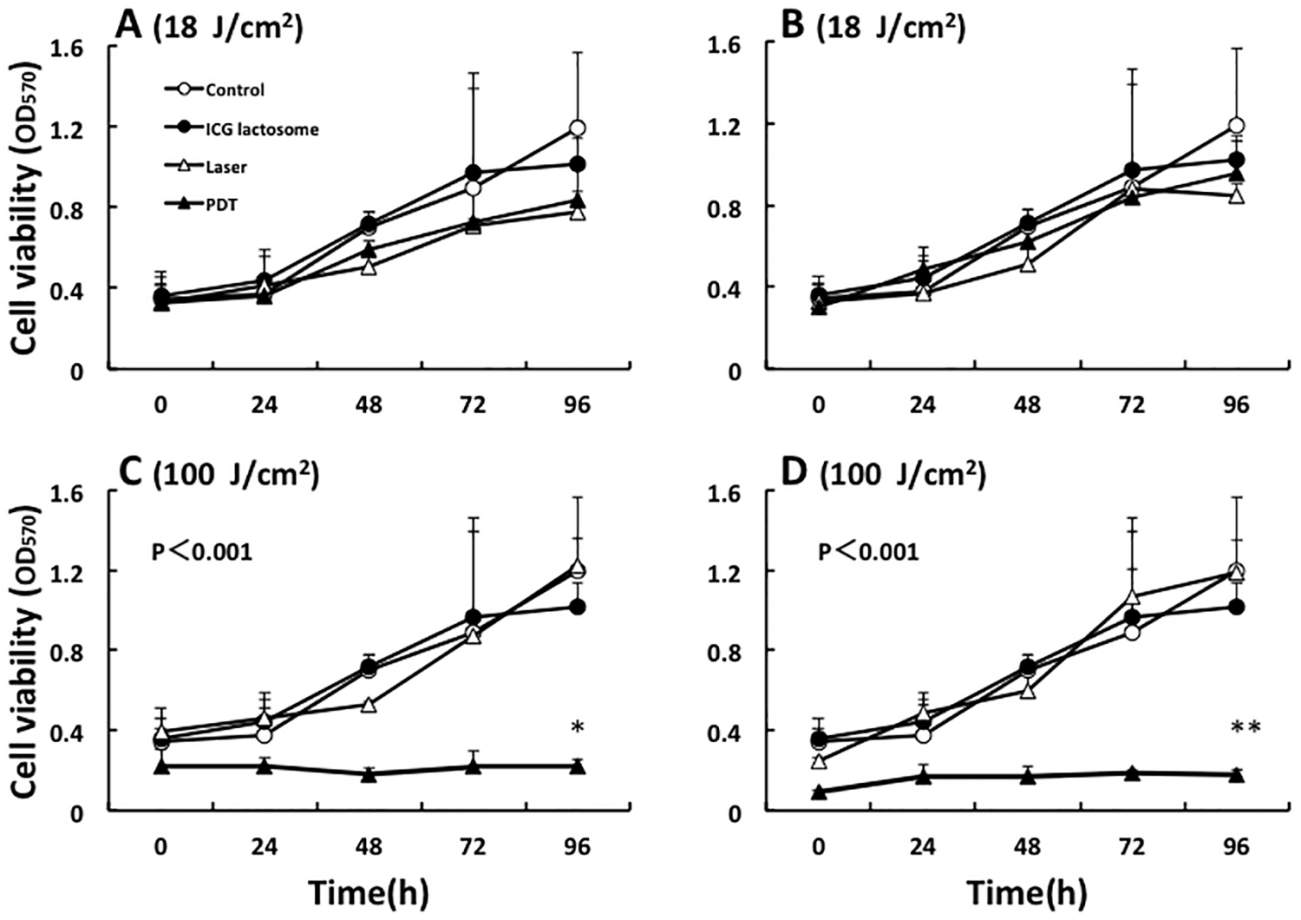
Effect of PDT on cell viability in ICG-lactosome-treated HuH-7 cells. Cells were divided into four groups: control, ICG-lactosome, laser, and PDT (ICG-lactosome + laser). The laser and PDT groups were irradiated with (A) 18 J/cm^2^ (190 mW/cm^2^ and 95 s), (B) 18 J/cm^2^ (340 mW/cm^2^ and 55 s), (C) 100 J/cm^2^ (190 mW/cm^2^ and 525 s), and (D) 100 J/cm^2^ (340 mW/cm^2^ and 300 s). Cell viability after irradiation was measured by the MTT assay at the indicated times (n = 4 dishes/time/group). *P<0.001 ICG-lactosome vs. other groups. OD_570_, optical density at 570 nm.

We examined the effects of laser irradiation with low or high fluence (18 or 100 J/cm^2^, respectively) on cell viability in ICG-lactosome-treated HuH-7 cells. Cells were divided into four groups: control; ICG-lactosome; laser; and PDT (ICG-lactosome + laser). We used four laser irradiation conditions: (A) 18 J/cm^2^ (190 mW/cm^2^ and 95 s); (B) 18 J/cm^2^ (340 mW/cm^2^ and 55 s); (C) 100 J/cm^2^ (190 mW/cm^2^ and 525 s); and (D) 100 J/cm^2^ (340 mW/cm^2^ and 300 s). At 96 h after irradiation, PDT groups with 100 J/cm^2^ (Fig 1C and 1D), but not 18 J/cm^2^ (Fig 1A and 1B), showed a marked decrease in cell viability compared with the control, ICG-lactosome, and laser groups.

**Fig 2:**
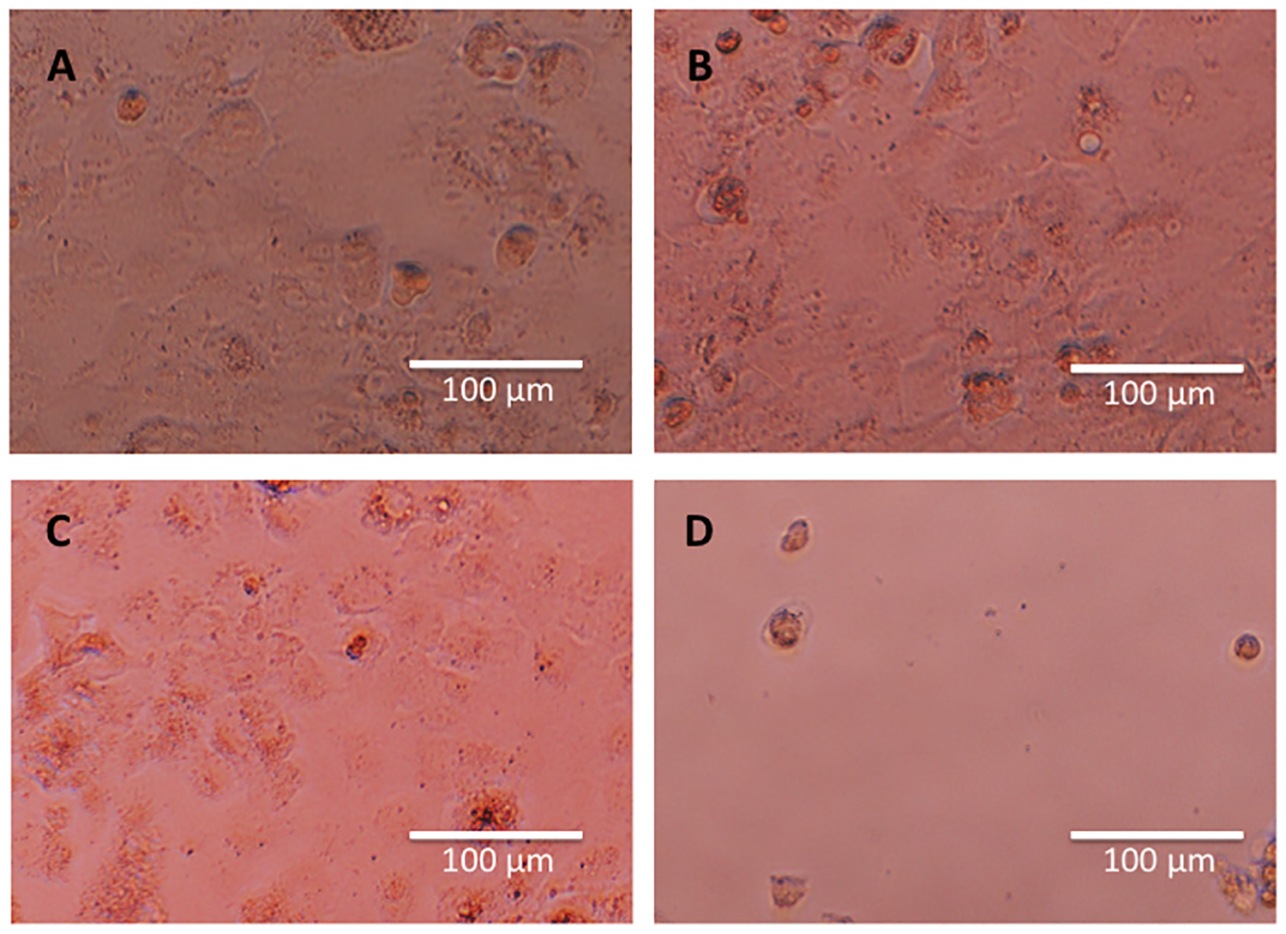
Effect of PDT on morphological changes in ICG-lactosome-treated HuH-7 cells. Morphological changes were observed with a phase-contrast microscope at 96 h after each treatment. (A) Control group. (B) ICG-lactosome group. (C) laser (100 J/cm^2^ (340 mW/cm^2^ and 300 s)) group. (D) PDT (ICG-lactosome + laser) group. Representative images from four experiments are shown.

Histological examination revealed a variety of changes in cell morphology when cells were subjected to ICG-lactosome and irradiation (PDT group). The cells became round and small in shape, with many cells floating in the culture medium, and the overall cell number was markedly decreased (Fig 2D). In contrast, there were neither obvious morphological changes nor cell number changes in the control, ICG-lactosome, and laser groups (Fig 2A–2C).

**Fig 3:**
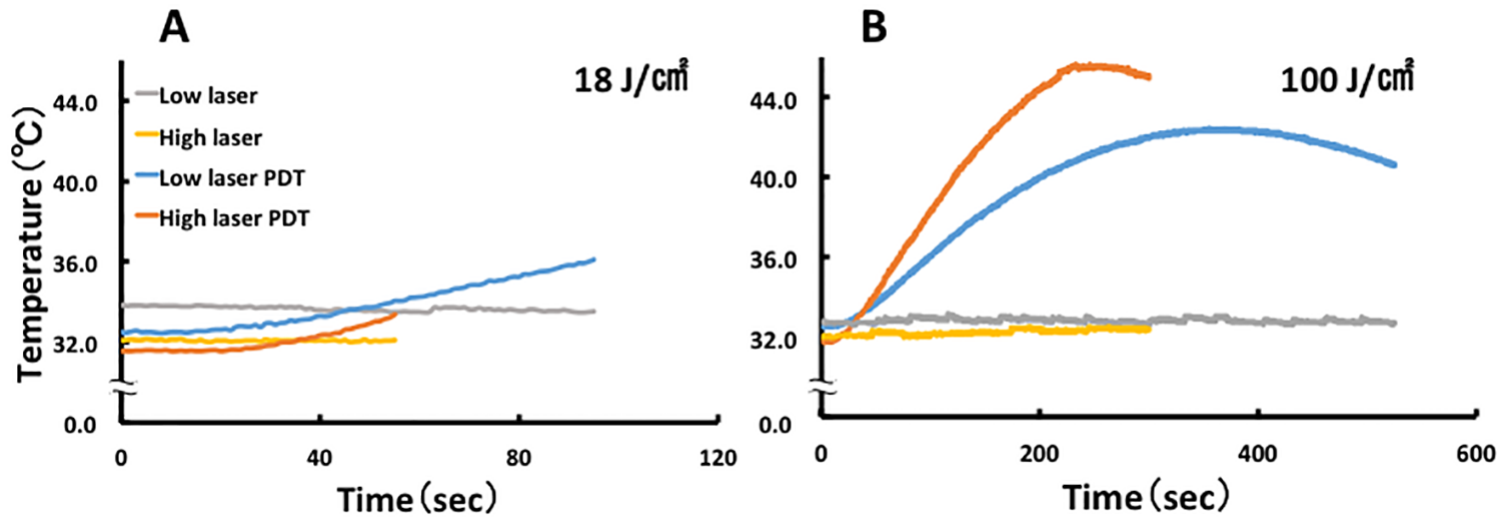
Effect of laser irradiation on temperature in ICG-lactosome-treated HuH-7 cells. The culture medium temperature was measured using a thermo-camera in the laser and PDT groups with low or high laser irradiation. (A) 18 J/cm^2^: low laser (190 mW/cm^2^ and 95 s), high laser (340 mW/cm^2^ and 55 s), low laser PDT, and high laser PDT (n = 4 dishes/group). (B) 100 J/cm^2^: low laser (190 mW/cm^2^ and 525 s), high laser (340 mW/cm^2^ and 300 s), low laser PDT. and high laser PDT (n = 4 dishes/group).

The effect of low and high laser treatment on cell temperature was compared between the laser and PDT groups. The temperature in all PDT groups increased. In contrast, no temperature increases were observed in the laser groups. In the PDT groups with 100 J/cm^2^ irradiation (Fig 3B), the temperature increased up to 42.0°C and 45.5°C for low and high power laser treatment, respectively, while lesser increases were observed in the PDT groups with 18 J/cm^2^ irradiation (Fig 3A).

### *In vivo* imaging

**Fig 4:**
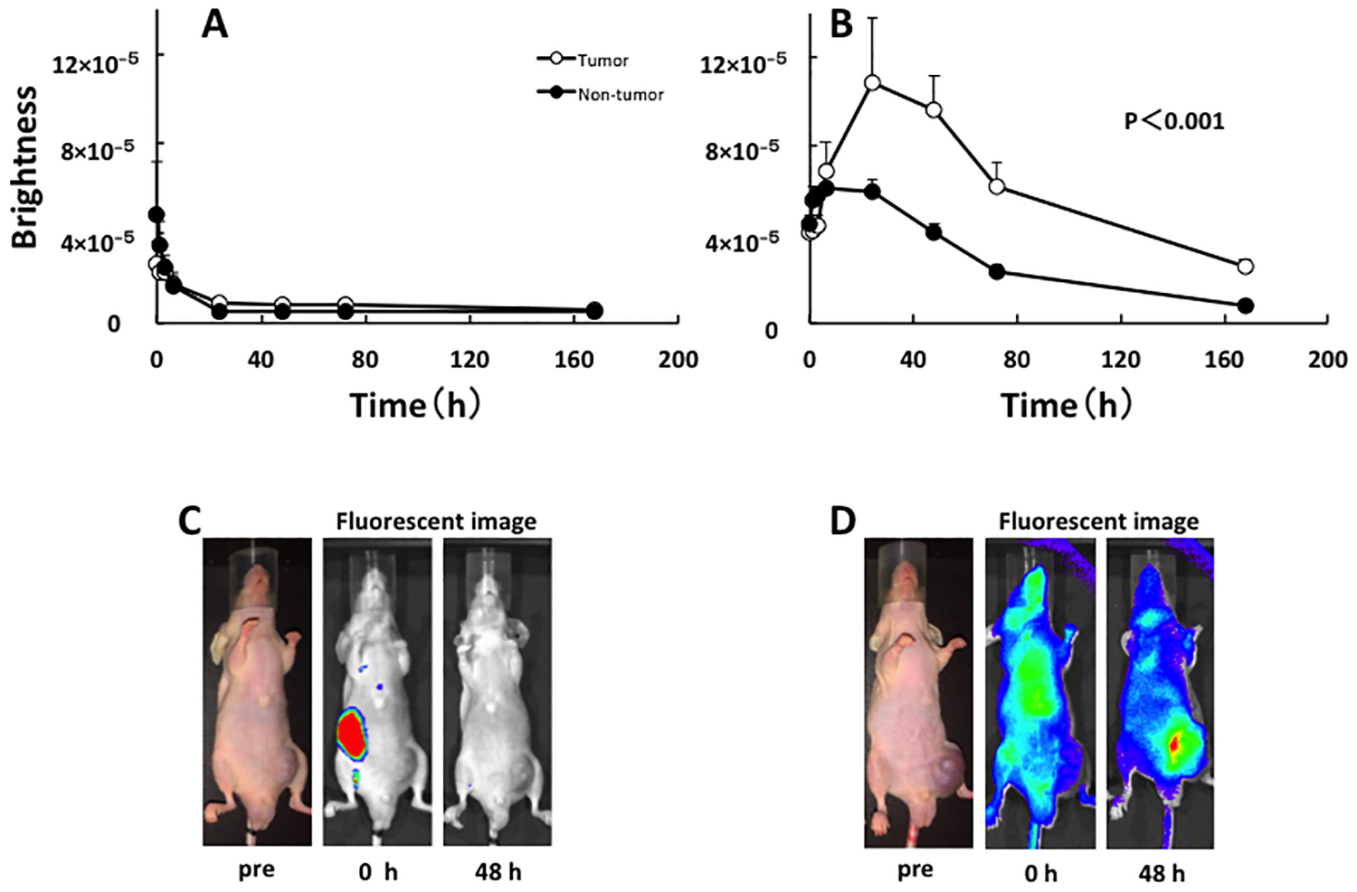
*In vivo* fluorescence imaging in the ICG-treated and ICG-lactosome-treated mice with subcutaneous tumors. Fluorescence imaging was acquired using an IVIS system in the ICG-treated mice (A and C) and ICG-lactosome-treated mice (B and D). After injection of ICG or ICG-lactosome, the brightness of the tumor (open circles) and non-tumor (contralateral inguinal, closed circles) areas was measured at the indicated times. P<0.001 between the tumor and non-tumor regions in the ICG-lactosome group; n = 5/group.

Using an IVIS system, *in vivo* fluorescence imaging was compared between the ICG and ICG-lactosome-treated mice with subcutaneous tumors. In the ICG-treated animals, ICG fluorescence accumulated in the liver just after the injection and migrated from the liver to the intestinal tract via bile duct exclusion within 1–6 h (Fig 4A and 4C). After 24 h, most of the ICG fluorescence was excluded from the body, although a minor portion remained in both the tumor and normal tissue areas. Comparing the brightness of tumor regions and normal tissue (contralateral inguinal) regions, no significant difference was noted between the two regions (Fig 4A).

In ICG-lactosome-treated animals, the fluorescence was distributed throughout the whole body just after the injection (Fig 4D). After 24–72 h, high levels of ICG fluorescence accumulated and remained in the tumor area, and there was a significant difference between the tumor and contralateral inguinal regions (Fig 4B and 4D). After 1 week, ICG fluorescence was still observed in the tumor area, while no fluorescence existed in the surrounding tissues or other organs (data not shown).

### *In vivo* PDT

**Fig 5:**
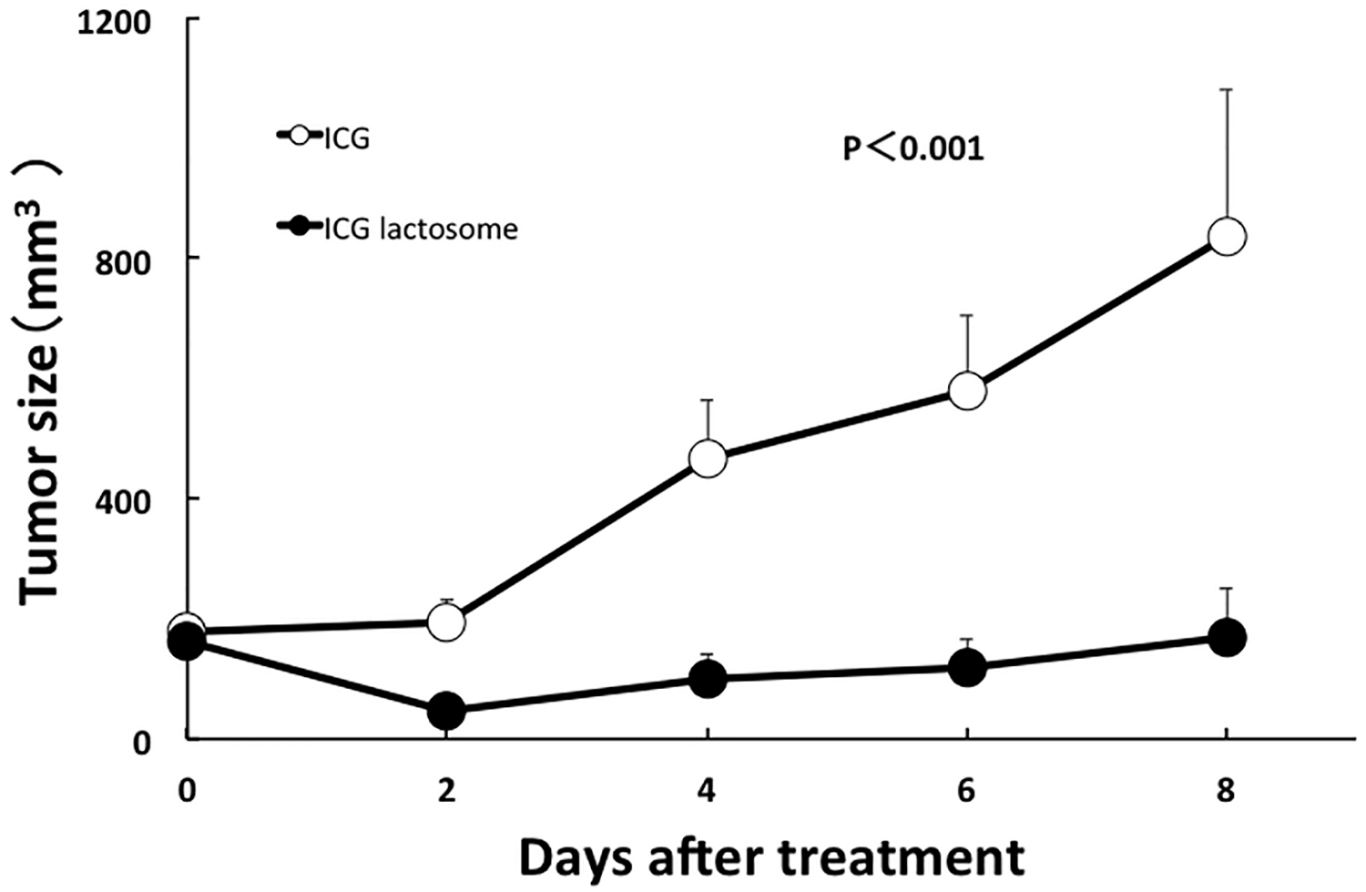
Effect of PDT on tumor growth in the ICG-treated and ICG-lactosome-treated mice with subcutaneous tumors. At 48 h after ICG (open circles) or ICG-lactosome (closed circles) injection in the tumor implanted-mice, laser irradiation (500 mW/cm^2^ and 200 s, 100 J/cm^2^) was carried out, and the tumor volumes were measured at the indicated times. P<0.001 between the ICG and ICG-lactosome groups; n = 6/group.

Tumor growth was compared between the ICG-treated and ICG-lactosome-treated mice with laser irradiation (n = 6/group). The mean tumor volumes in the ICG group markedly increased from day 0 (176.6±29.0 mm^3^) to day 8 (834.3±244.9 mm^3^). In contrast, the mean tumor volumes in the ICG-lactosome group showed no significant growth from day 0 (162.9±21.4 mm^3^) to day 8 (167.8±81.1 mm^3^). There was a significant difference between the two groups (Fig 5).

**Fig 6:**
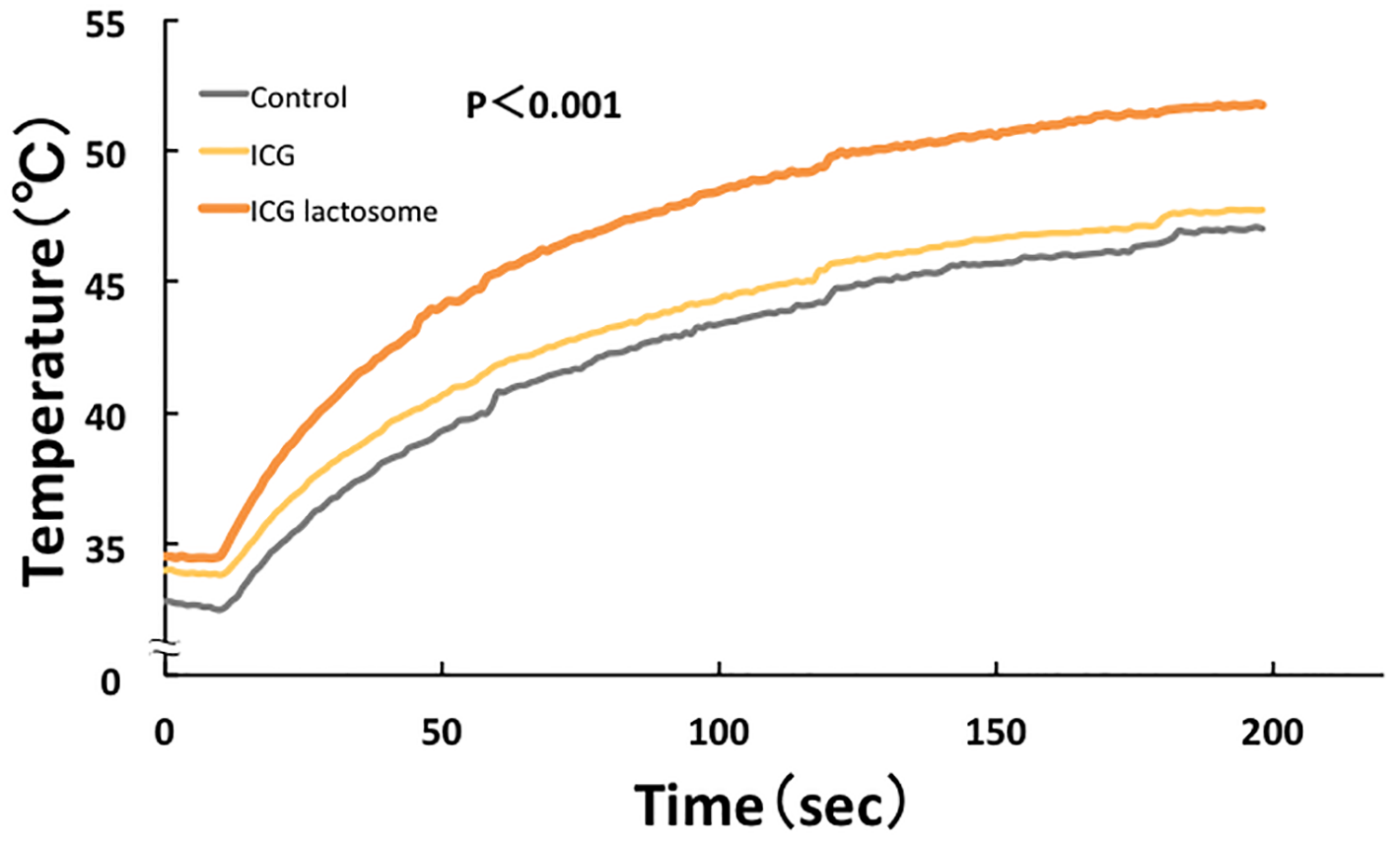
Effect of laser irradiation on tumor temperature in the ICG-treated and ICG-lactosome-treated mice with subcutaneous tumors. At 48 h after ICG (yellow) or ICG-lactosome (brown) injection or no injection (control, dark) in the tumor implanted-mice, the laser irradiation (500 mW/cm^2^ and 200 s, 100 J/cm^2^) treatment was initiated, and the temperatures were measured at the indicated times. P<0.001 between the ICG and ICG-lactosome groups; n = 6/group.

**Fig 7:**
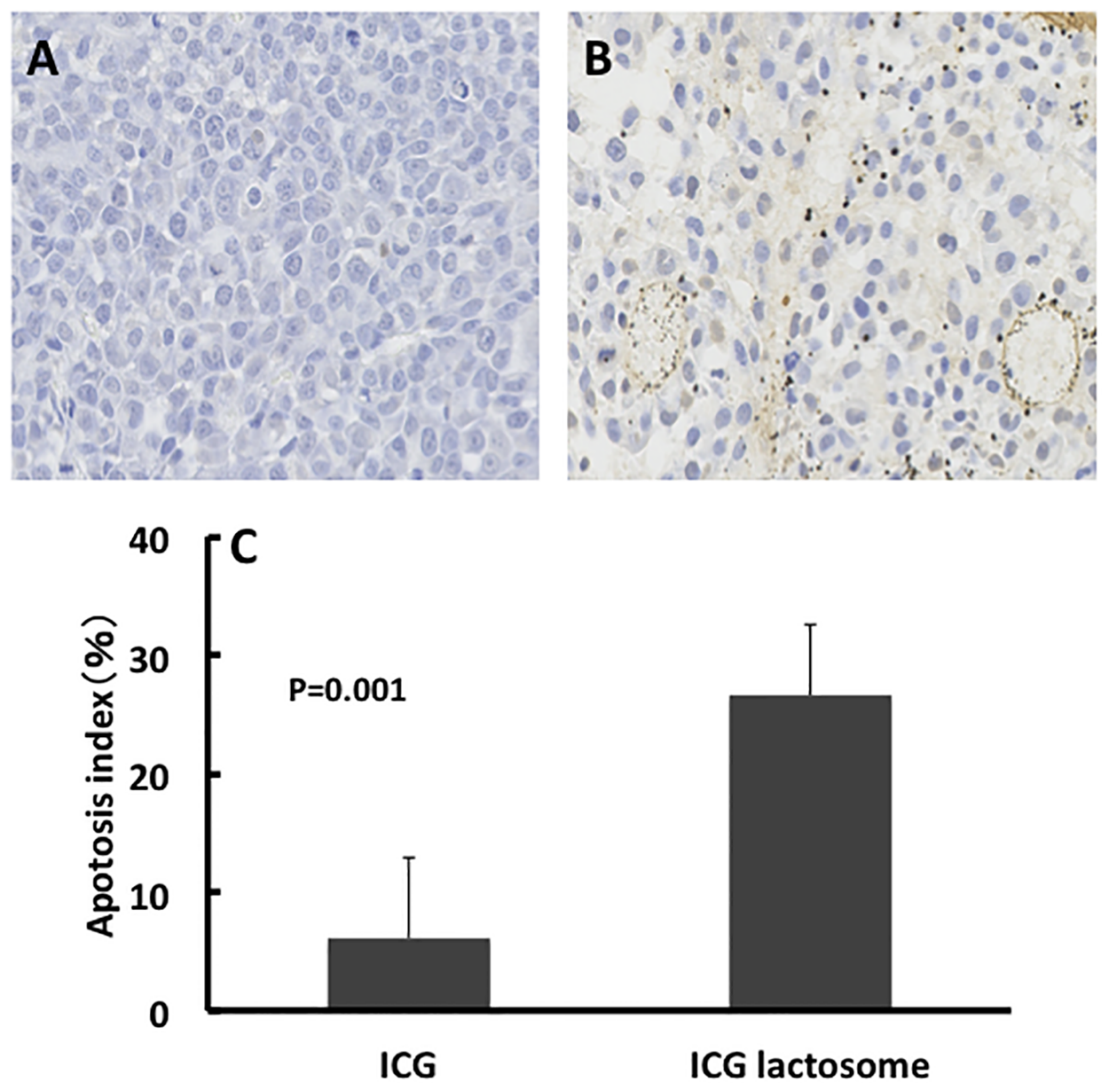
Effect of PDT on cells of subcutaneous tumors in the ICG-treated and ICG-lactosome-treated mice. Tumors were obtained for examination of apoptosis at 24 h after irradiation (500 mW/cm^2^ and 200 s, 100 J/cm^2^) in the ICG (A) and ICG-lactosome (B) groups. TUNEL staining was performed on the specimens. (C) Apoptotic indexes in the ICG-treated and ICG-lactosome-treated mice with subcutaneous tumors. P = 0.001 between the ICG and ICG-lactosome groups; n = 5/group.

During laser irradiation, the temperature in the tumor area increased from 34.0°C to 47.7°C and from 34.5°C to 51.7°C at 0–200 s in the ICG and ICG-lactosome groups, respectively (Fig 6). The temperature in the ICG-lactosome group was higher than that in the ICG group (P<0.001). In the ICG-lactosome group, substantial apoptotic cell death was seen, and the apoptotic index was significantly higher in the ICG-lactosome group than in the ICG group (Fig 7, P = 0.001).

## Discussion

Using ICG-lactosome, investigators can diagnose or treat a variety of tumors by fluorescence imaging or PDT, respectively. Funayama et al. [24–26] reported that ICG-lactosome selectively accumulated in spinal metastasis, and that PDT using ICG-lactosome delayed the development of paralysis in a rat spinal metastasis model. Tsujimoto et al. [23,27] reported a comparison of the diagnostic and treatment effects of ICG and ICG-lactosome. Although ICG-lactosome selectively accumulated in lymph node metastases and peritoneal disseminations of gastric cancer, no accumulation in the tumor was observed with ICG. Furthermore, PDT using ICG-lactosome significantly inhibited the growth of metastases compared with PDT with ICG. In this study, *in vivo* imaging demonstrated that ICG-lactosome, but not ICG alone, was specifically incorporated and clearly visualized in subcutaneous xenograft tumors of human HCC. In addition, we found both *in vitro* and *in vivo* that PDT using ICG-lactosome induced apoptosis and inhibited tumor growth. These results are probably derived from selective accumulation of ICG in tumors by ICG-lactosome.

We identified tumors using ICG-lactosome in xenografts of human HCC. In HuH-7 cells, administration of high-dose ICG results in accumulation of ICG in tumors. However, the concentration of ICG used in this study was low compared with those in other studies [10]. For this reason, it is considered that the ICG accumulation in the tumors was small, and fluorescence could not be obtained. In that regard, as ICG-lactosome selectively accumulated in tumors through the EPR effect, we considered that the tumors could be detected even with the same ICG concentration. The fact that an antitumor effect of PDT was not obtained in the ICG group could also be attributed to insufficient accumulation of ICG in the tumors. These considerations suggest that increased amounts of ICG-lactosome would facilitate the visualization of more tumors, including small ones.

The presumed mechanism of tumor suppression by PDT using ICG-lactosome involves singlet oxygen and ICG degradation products generated by a photochemical reaction as reported previously [9]. In PDT, when the photosensitizing agent is exposed to light of a specific wavelength, it is activated from the ground state to an excited state. As it returns to the ground state, it releases energy, which is transferred to oxygen molecules to generate ROS, such as singlet oxygen and free radicals, and it is these ROS that mediate cellular toxicity. Similarly, when ICG is exposed to near-infrared excitation light, singlet oxygen can be generated. ICG is degraded by the singlet oxygen itself, and the decomposition products further decrease cell viability [28]. These products may lead to tumor cell apoptosis.

However, heat generated by a photothermal reaction was markedly involved in the tumor suppression by PDT using ICG-lactosome. The temperature measured during *in vivo* PDT with ICG-lactosome showed a substantial increase (50°C and above) compared with ICG. This suggests that the heat may be sufficient to damage tumor cells, followed by the involvement of tumor growth suppression. In addition to thermal regulation, it is necessary to optimize the ICG-lactosome dosage as well as the fluence of laser irradiation to prevent future tumor growth.

We believe that the temperature increase is one of the main factors in the antitumor effects obtained in our study, because it was previously reported that PDT using ICG also had a thermal effect [29]. It seems likely that the temperature increase by PDT depends on a variety of photosensitizers including ICG. PDT using ICG gives rise to a temperature increase that leads to antitumor effects, thereby indicating the usefulness of ICG-lactosome.

In conclusion, the present results suggest that ICG-lactosome is useful as a novel diagnostic and therapeutic agent for HCC, similar to the case for peritoneal dissemination in gastric cancer [23], spinal metastasis [24–26], lymph node metastases in gastric cancer [27], and metastatic bone tumors in human breast cancer [30]. PDT using ICG-lactosome can be repeatedly performed and can be used in endoscopic or laparoscopic therapy for small HCC. As future prospects, we will optimize drug dosage and irradiation conditions, and examine the effect of treatment on other carcinomas to develop this treatment into a clinical application.
